# *fs(1)h* controls metabolic and immune function and enhances survival via AKT and FOXO in *Drosophila*

**DOI:** 10.1242/dmm.037259

**Published:** 2019-04-04

**Authors:** Jessica Sharrock, Alicia Estacio-Gomez, Jake Jacobson, Katrin Kierdorf, Tony D. Southall, Marc S. Dionne

**Affiliations:** 1 MRC Centre for Molecular Bacteriology and Infection, Imperial College London, London SW7 2AZ, UK; 2 Department of Life Sciences, Imperial College London, London SW7 2AZ, UK

**Keywords:** *Drosophila melanogaster*, Antimicrobial peptide, Bromodomain protein, Insulin

## Abstract

The *Drosophila* fat body is the primary organ of energy storage as well as being responsible for the humoral response to infection. Its physiological function is of critical importance to the survival of the organism; however, many molecular regulators of its function remain ill-defined. Here, we show that the *Drosophila melanogaster* bromodomain-containing protein FS(1)H is required in the fat body for normal lifespan as well as metabolic and immune homeostasis. Flies lacking fat body *fs(1)h* exhibit short lifespan, increased expression of immune target genes, an inability to metabolize triglyceride, and low basal AKT activity, mostly resulting from systemic defects in insulin signalling. Removal of a single copy of the AKT-responsive transcription factor *foxo* normalises lifespan, metabolic function, uninduced immune gene expression and AKT activity. We suggest that the promotion of systemic insulin signalling activity is a key *in vivo* function of fat body *fs(1)h*.

This article has an associated First Person interview with the first author of the paper.

## Abstract (precis)

**Summary:** The bromodomain-containing protein FS(1)H is required in the *Drosophila* fat body for normal lifespan and metabolic and immune function, largely via the insulin pathway.

## INTRODUCTION

The *Drosophila* fat body is the primary site of energy storage as well as being the source of the transcriptionally induced humoral immune response (Lemaitre and Hoffmann, 2007). *Drosophila* immune responses involve several interdependent effector mechanisms, including melanisation, phagocytosis and transcriptional induction of antimicrobial peptides (AMPs) (Lemaitre and Hoffmann, 2007). AMP transcriptional induction is elicited by detection of bacterial peptidoglycan, fungal glucans or other mechanisms (Aggrawal and Silverman, 2007; Gottar et al., 2006). The Toll and immune deficiency (IMD) pathways are the two known peptidoglycan-responsive signalling pathways in *Drosophila*. These pathways respond to discrete microbial moieties to activate the expression of AMPs via distinct NF-κB transcription factors (Lemaitre and Hoffmann, 2007). AMPs are then secreted into the haemolymph, where they are able to directly kill invading microorganisms. Importantly, although signalling via these NF-κB pathways is critical for induction of AMPs in response to systemic infection, several other transcription factors are important regulators of AMPs in the absence of infection and in other tissues – in particular, in barrier epithelia (Becker et al., 2010; Clark et al., 2013; Junell et al., 2010; Reed et al., 2008; Rynes et al., 2012; Senger et al., 2006).

In addition to these immune roles, the fat body is analogous to vertebrate adipose tissue and liver, and functions as an important organ in nutrient storage and energy metabolism (Arrese and Soulages, 2010). As the primary location of metabolic stores, the fat body is critical for normal metabolic homeostasis in the fly when food is scarce (Arrese and Soulages, 2010). This organ contains both glycogen and triglyceride; their storage and release is controlled by various endocrine pathways. The most prominent of these pathways in promotion of anabolism is the insulin signalling pathway. Insulin signalling is evolutionarily conserved in animals, and plays important roles in regulating metabolism, controlling growth and determining lifespan (Taguchi and White, 2008). In *Drosophila*, there are eight insulin-like peptides (ILPs), of which three – ILP2, ILP3 and ILP5 – are believed to play roles analogous to that played by insulin in regulating physiology in response to transient changes in nutritional state (Broughton et al., 2005; Ikeya et al., 2002; Rulifson et al., 2002). These ILPs are released from insulin-producing cells (IPCs) in the brain. Other ILPs, with sometimes-overlapping physiological effects, can be produced by the fat body (Slaidina et al., 2009), the ventral nerve cord (Brogiolo et al., 2001) and other tissues (Colombani et al., 2012; Garelli et al., 2012). These ILPs signal via a conserved PI3K pathway to activate the serine-threonine kinase AKT; the best-known transcriptional effects of this pathway involve the signal-responsive inactivation of the transcription factor FOXO (Jünger et al., 2003; Kramer et al., 2003). In recent years, several observations have begun to shed light on how the distinct physiological functions of the fat body in nutrient storage and the immune response can be integrated and regulated in a single tissue (Becker et al., 2010; Clark et al., 2013; Rynes et al., 2012; Varma et al., 2014). However, there are still significant gaps in our knowledge of these mechanisms (Dionne, 2014).

Bromodomain-containing proteins (BCPs) are well characterised as regulators of transcription. The bromodomain, originally described as a domain common to *Drosophila* BRM and yeast SNF2, binds acetylated lysine residues (Dhalluin et al., 1999; Tamkun et al., 1992). In transcriptional contexts, the bromodomain binds acetylated lysines in histone tails to facilitate recruitment of transcription factors and other chromatin-binding proteins (Wu and Chiang, 2007). In part due to their general role in transcriptional activation, BCPs are associated with a wide variety of biological processes (Houzelstein et al., 2002; Mishima et al., 2011; Shang et al., 2007, 2009; Velisek et al., 2011; Wang et al., 2009). *female sterile (1) homeotic* (*fs(1)h*) encodes the sole *Drosophila* member of the bromodomain and extra-terminal motif (BET) protein family, homologous to mammalian BCPs BRD2/3/4/T (Florence and Faller, 2001). These proteins are characterised by two bromodomains and an adjacent extra-terminal, or ET, domain. Like other BCPs, FS(1)H is able to recognise and bind acetylated lysine residues, as well as interact with and function as a scaffold for molecules implicated in gene transcription (Sanchez and Zhou, 2009). In recent years, pharmacological inhibitors of BET family proteins have been suggested as treatments for inflammation and autoimmune disease (Bandukwala et al., 2012; Mele et al., 2013; Nicodeme et al., 2010). However, the broader *in vivo* roles of BET domain proteins in immune regulation are not entirely clear.

We show here for the first time that *fs(1)h*, the sole *Drosophila* BET-family protein, is required in the fat body for normal lifespan and immune and metabolic function. Flies with fat-body-specific *fs(1)h* knockdown exhibit abnormally high expression of AMPs, whether uninfected or infected with bacteria; low levels of free sugar, combined with an inability to utilise triglyceride; and an extremely short lifespan. *fs(1)h* knockdown animals also exhibit significantly reduced systemic AKT activity, which is the underlying cause of nearly all these observed phenotypes. This effect may result from increased expression of several secreted ILP antagonists. Removal of a single copy of the AKT-responsive transcription factor *foxo* ameliorates almost all of the observed phenotypes, restores the lifespan of these animals to normalcy and normalises expression of insulin-regulatory signals, suggesting that much of the *in vivo* activity of *fs(1)h* in this tissue is mediated via *foxo* regulation.

## RESULTS

### Loss of *fs(1)h* in the fat body shortens lifespan

**Fig. 1.**
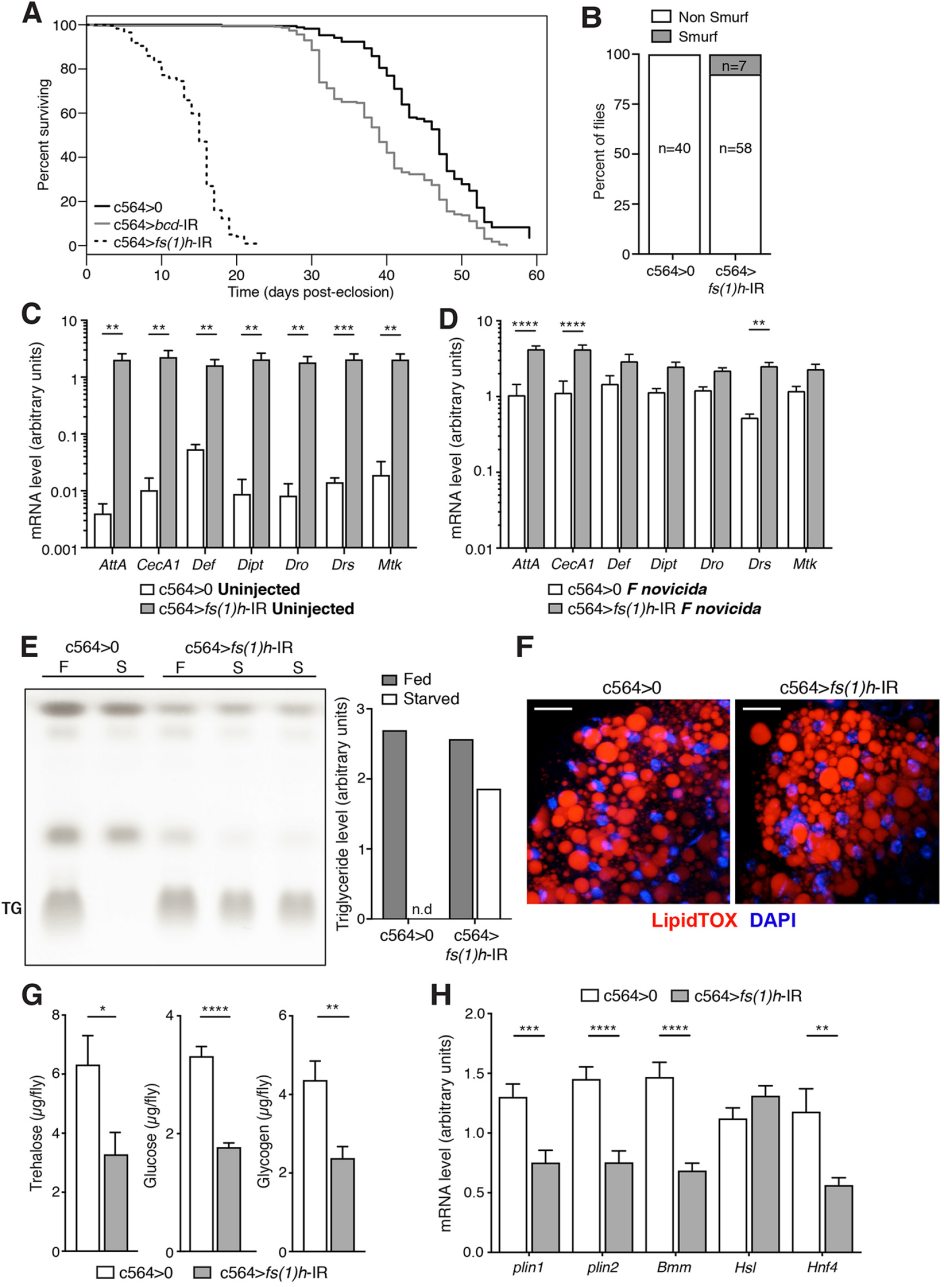
***fs(1)h* is required in fat body for normal lifespan, immune gene expression and physiology.** (A) Survival of male flies with fat-body *fs(1)h* knockdown (c564>*fs(1)h*-IR), an irrelevant knockdown (c564>*bcd*-IR) and driver-only controls (c564>0) at 25°C. (Here and in all other cases, flies are on a *w^1118^* genetic background; ‘c564>0’ indicates the driver-only control line, in which Gal4 is expressed but no knockdown is present.) Number of flies: c564>0, *n*=178; c564>*bcd*-IR, *n*=235; c564>*fs(1)h*-IR, *n*=230. (B) Smurf assay of gut integrity in 5- to 7-day-old *fs(1)h* knockdowns and wild-type controls. Number of flies: c564>0, *n*=40; c564>*fs(1)h*-IR, *n*=65. Values are statistically different by Fisher's exact test (*P*=0.0424). (C,D) Expression of antimicrobial peptide (AMP) genes in isolated fat body from 5- to 7-day-old male control (c564>0) and fat-body *fs(1)h* knockdown (c564>*fs(1)h-IR*) flies. Normalised to expression of α-tubulin as a loading control; here and elsewhere, graphed qRT-PCR values are these tubulin-normalised abundance measurements. Values shown as mean+s.e.m. (C) Uninjected flies. (D) *Francisella novicida*-injected flies. (E) Triglyceride (TG) levels in control and fat-body *fs(1)h* knockdown flies under normal conditions and following 24-h starvation (F, fed; S, starved; n.d., no TG detected). Values shown as mean. (F) Isolated fat-body tissue from *fs(1)h* knockdowns and controls, stained with LipidTOX (red; TG) and DAPI (blue; nuclei). Scale bars: 20 μm. Representative of eight flies per genotype. (G) Free sugar (trehalose and glucose) and stored carbohydrate (glycogen) levels in controls and *fs(1)h* fat-body-knockdown flies. Values represent six replicates per genotype. Shown as mean+s.e.m.; genotypes were compared using unpaired two-tailed *t*-test. (H) Expression of *plin1*, *plin2*, *Bmm, Hsl* and *Hnf4* in isolated fat body from control and *fs(1)h* fat-body-knockdown flies. Normalised to expression of α-tubulin as a loading control. Values shown as mean+s.e.m.; genotypes were compared using unpaired, two-tailed *t*-test. 8-10 samples per genotype. Throughout, *P<0.05, **P<0.01, ***P<0.001, ****P<0.0001.

A small RNAi screen of BCPs for factors involved in infection survival revealed that flies with *fs(1)h* knocked down in the fat body exhibited dramatically reduced survival even when uninfected (Fig. 1A, Fig. S1A). This phenotype was equally strong with a second, non-overlapping *fs(1)h* dsRNA (Fig. S1B) and with r4-Gal4, a second fat-body driver (Fig. S1C) (Lee and Park, 2004). Gut barrier dysfunction, as measured by the Smurf assay, has previously been reported as a predictor of death in flies dying of old age, although the causal importance of this correlation is not always clear (Rera et al., 2012). In keeping with their short lifespan, a larger fraction of *fs(1)h* knockdown flies exhibited gut barrier dysfunction than the control genotype, although not necessarily to the levels that have been reported for wild-type *w^1118^* flies dying of old age (Fig. 1B).

### *fs(1)h* loss in the fat body increases antimicrobial peptide expression

The fat body is the primary site of the humoral immune response in *Drosophila* (Arrese and Soulages, 2010). AMPs are the most well-characterised of the genes transcriptionally induced by infection; they are produced in the fat body and secreted into the haemolymph (Lemaitre and Hoffmann, 2007). Therefore, we assayed fat body immune function in *fs(1)h* knockdown flies by testing expression of AMPs. We observed significant increases in AMP expression in *fs(1)h* knockdown animals (Fig. 1C,D, Fig. S1D). This effect was also present in uninfected flies as well as in flies that had been infected with bacteria, and was observed for most AMPs tested, including targets of both Toll and IMD pathways (e.g. *Drosomycin* and *Diptericin*, respectively). This indicates that *fs(1)h* is an endogenous negative regulator of AMP expression, acting in the presence and absence of infection.

To determine whether known immune signalling pathways were involved in the increased AMP expression observed in *fs(1)h* knockdowns, we assayed REL protein expression and processing (Stöven et al., 2000); REL is the NF-κB family member responsible for IMD-dependent AMP expression (Fig. S1E). We found that total REL abundance was dramatically increased in *fs(1)h* knockdowns, but the quantity of the C-terminal cleavage product, indicative of pathway activation, was not different between wild-type controls and *fs(1)h* knockdowns; this was true whether the flies were infected or uninfected. This suggests that the increase in baseline AMP expression does not reflect an overall increase in IMD pathway activity.

### Flies lacking fat body *fs(1)h* fail to use triglyceride stores and are hypoglycaemic

In addition to its role in immunity, the fat body is the primary location of triglyceride and glycogen storage and a key gluconeogenic tissue. We next assayed metabolic stores and free sugar levels in *fs(1)h* knockdown flies.

The quantity of triglyceride in undisturbed fat-body *fs(1)h* knockdowns was heterogeneous; we sometimes observed essentially normal triglyceride levels, while at other times triglycerides were elevated roughly twofold. However, we consistently observed that, when starved, *fs(1)h* knockdowns were unable to utilize their triglyceride stores (Fig. 1E). Microscopic analysis of adult fat body revealed no overt morphological abnormality in the tissue (Fig. 1F). *fs(1)h* knockdown also affected carbohydrate metabolism: in these animals, we observed reductions in glycogen (stored carbohydrate) as well as in free glucose and trehalose (circulating sugars) (Fig. 1G). These phenotypes were reminiscent of those seen in mice heterozygous for *Brd2* mutation, which are obese and exhibit reduced serum glucose (Wang et al., 2009).

Having identified impaired utilisation of triglycerides in these flies, we measured the transcript levels of key activities in triglyceride utilization, including the lipases *brummer* (*bmm*) and *Hormone sensitive lipase* (*Hsl*) and the perilipins *Plin1* and *Plin2*. Of these genes, the perilipins and *bmm* were reduced in expression, although not eliminated (Fig. 1H). Finally, we assayed the expression of the nuclear receptor *Hnf4*, a key transcriptional regulator of triglyceride and glucose metabolism, and observed a significant reduction in its expression (Fig. 1H); because HNF4 transcriptional activity is regulated by long-chain fatty acids, it is not clear whether this reduced expression causes a corresponding reduction of HNF4 transcriptional activity (Barry and Thummel, 2016; Palanker et al., 2009; Storelli et al., 2019).

### Fat-body *fs(1)h* knockdown may reduce AKT activation by inducing expression of ILP antagonists

**Fig. 2.**
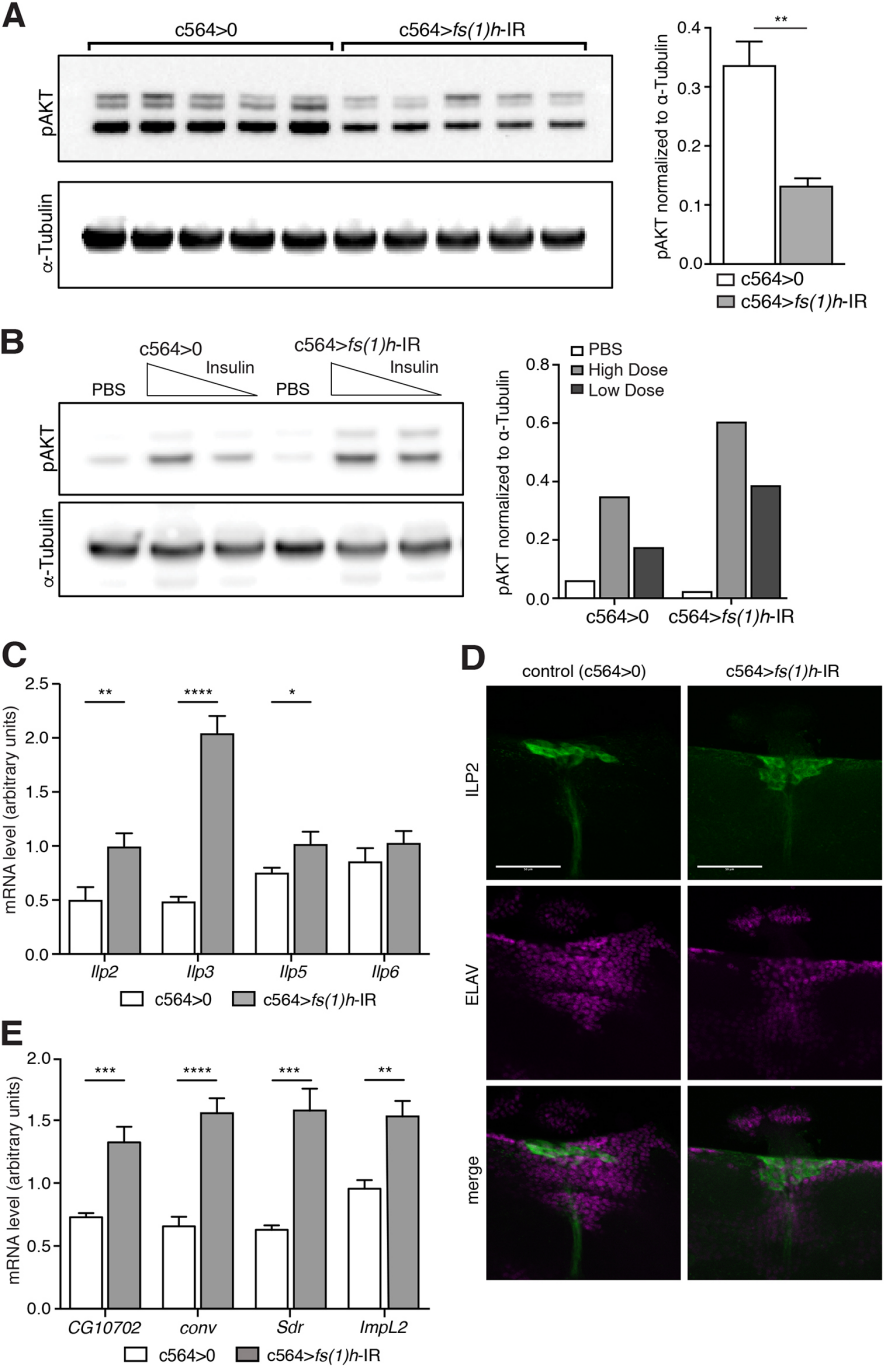
**Fat body *fs(1)h* promotes AKT activity.** (A) Western blot analysis of AKT Ser505 phosphorylation in control and *fs(1)h* fat-body-knockdown flies. Values represented as intensity relative to α-tubulin, shown as mean+s.e.m. (**P<0.01). (B) Western blot analysis of AKT Ser505 phosphorylation following injection with PBS or a high (320 pg/fly) or low (64 pg/fly) dose of human insulin, to assay insulin sensitivity. Values relative to α-tubulin, shown as mean. (C) Expression of *Ilp2*, *Ilp3*, *Ilp5* and *Ilp6* by qRT-PCR in whole control and *fs(1)h* fat-body-knockdown flies, shown as mean+s.e.m. Normalised to expression of α-tubulin as a loading control. (D) Immunostaining for ILP2 (green) and ELAV (magenta; labels all neurons; used as a counterstain). Scale bars: 50 μm. (E) Expression of *CG10702*, *conv*, *Sdr* and *ImpL2* by qRT-PCR in whole control and *fs(1)h* fat-body-knockdown flies, shown as mean+s.e.m.; genotypes were compared using unpaired, two-tailed *t*-test. Normalised to expression of α-tubulin as a loading control. Ten samples per genotype. Throughout, *P<0.05, **P<0.01, ***P<0.001, ****P<0.0001.

The insulin signalling pathway has conserved roles in the regulation of metabolism and lifespan, and can also affect AMP expression (Becker et al., 2010; Taguchi and White, 2008). Based on our phenotypic observations, we assayed insulin pathway activity in *fs(1)h* knockdowns. AKT is the key effector kinase of the insulin signalling pathway; its activity is stimulated by phosphorylation at serine 505 (Ser505). We found a significant reduction in levels of phospho-Ser505 AKT in *fs(1)h* knockdowns relative to controls (Fig. 2A). Again, this phenotype was present in flies with a second, non-overlapping *fs(1)h* knockdown, and was also visible (although more weakly) with r4-Gal4, a second fat-body driver (Fig. S2A,B).

Changes in systemic AKT activity might result from changes in the production or secretion of ILPs, changes in the expression of molecules that bind ILPs in the extracellular space and prevent them from signalling, or changes in the inherent ability of peripheral tissues to respond to ILPs (‘insulin resistance’). We tested our flies for insulin resistance by testing their response to injected human insulin. We found that *fs(1)h* knockdowns were perfectly capable of activating AKT in response to exogenous insulin (Fig. 2B). This indicated that the difference must be upstream of detection by the insulin receptor, either in the production or secretion of ILPs from their source cells, or in the production of ILP-antagonistic proteins that could not interact with human insulin.

We next tested the production and secretion of ILPs. We assayed expression of the primary metabolically relevant ILPs by quantitative reverse-transcription PCR (qRT-PCR) and found either small changes, or elevated expression, indicating that *fs(1)h* knockdown was not directly inhibiting ILP expression (Grönke et al., 2010) (Fig. 2C). We then tested the expression of factors known to regulate the secretion of ILPs, but we found heterogenous effects on these genes (Fig. S2C) (Koyama and Mirth, 2016; Rajan and Perrimon, 2012; Sano et al., 2015). To resolve the importance of this, we assayed the secretion of ILPs from median neurosecretory cells by immunostaining with an anti-ILP2 antibody; defects in ILP secretion are often observed as an increase in accumulation of intracellular ILP2 (Rajan and Perrimon, 2012). We observed no consistent difference in ILP2 immunoreactivity in these cells, indicating that ILP secretion was functioning more-or-less normally in *fs(1)h* knockdowns (Fig. 2D).

These observations together indicated that the observed defect in AKT activity might be caused by increased expression of one or more extracellular antagonists of ILP signalling. We assayed the expression of the characterised ILP antagonists *conv*, *Sdr* and *ImpL2*, as well as *CG10702*, which encodes an apparent insulin receptor homologue lacking the kinase domain and thus might also function as an ILP antagonist (Arquier et al., 2008; Honegger et al., 2008; Okamoto et al., 2013). All four ILP antagonists were strongly elevated in *fs(1)h* knockdowns (Fig. 2E), suggesting that the defect in physiological AKT activity might be generated, at least in part, by overexpression of these extracellular ILP antagonists. This was consistent with the strength of the observed loss in phospho-AKT, which was stronger than we would have expected from a fat-body-specific effect, especially in flies with *fs(1)h* knocked down by the GD51227 line (Fig. S2A).

### Increased FOXO activity drives many aspects of the *fs(1)h* knockdown phenotype

**Fig. 3.**
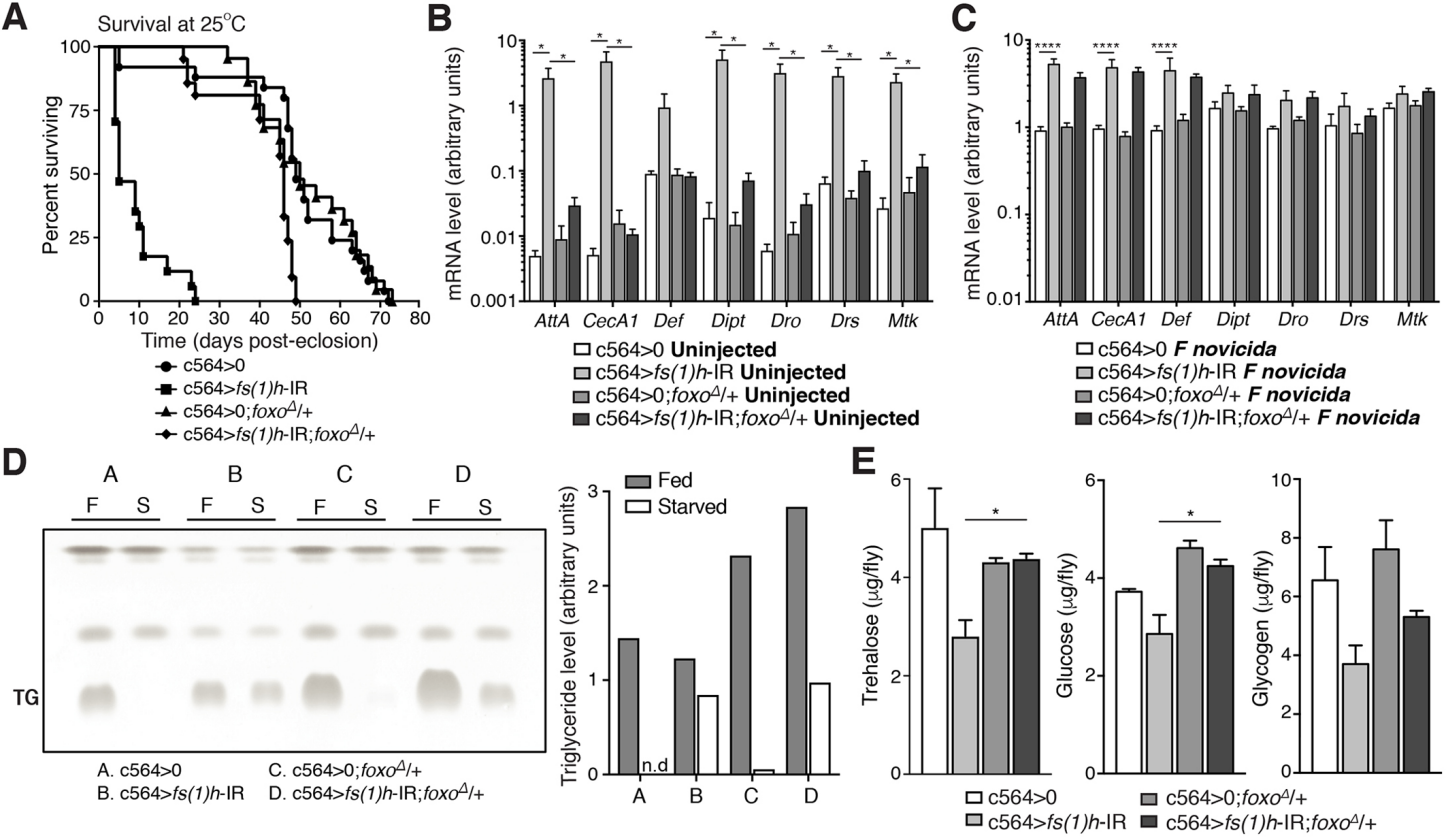
**Increased FOXO activity drives pathophysiology in fat body *fs(1)h* knockdowns.** (A) Survival of male *fs(1)h* knockdowns and controls, wild-type or heterozygous for *foxo*, at 25°C. Number of flies in this experiment: 30-40 per genotype. (B,C) Expression of antimicrobial peptide (AMP) genes in control (c564>0) and *fs(1)h* knockdown (c564>*fs(1)h*-IR) flies, either wild-type or heterozygous for *foxo*. Values represent mean+s.e.m.; genotypes were compared using ANOVA (*P<0.05, ****P<0.0001). (B) Isolated fat body, uninjected flies. (C) *Francisella novicida*-injected whole flies. (D) Triglyceride (TG) levels in control and *fs(1)h* fat-body-knockdown flies, either wild-type or heterozygous for *foxo*, under normal conditions and following 24 h starvation (F, fed; S, starved; n.d., no TG detected). Values shown as mean. (E) Free sugar (trehalose and glucose) and stored carbohydrate (glycogen) levels in control and *fs(1)h* fat-body-knockdown flies, either wild-type or heterozygous for *foxo*. Values as mean+s.e.m.; genotypes were compared using unpaired two-tailed *t*-test (*P<0.05). Number of samples: six replicates per genotype.

The best-characterised transcriptional effector downstream of AKT is FOXO. FOXO is phosphorylated by AKT, promoting its nuclear exclusion, so that reducing AKT activity should increase FOXO activity. To determine the extent to which FOXO hyperactivation was responsible for the phenotypes of *fs(1)h* knockdown flies, we combined *fs(1)h* knockdown with a loss-of-function mutation in *foxo*. Remarkably, we observed that heterozygosity for a *foxo*-null allele was sufficient to completely rescue the lifespan defect of *fs(1)h* knockdown flies (Fig. 3A). This could be explained if *foxo* were required for the expression of *Gal4*, so that *foxo* mutant animals would not express the knockdown. We tested this by assaying *Gal4* expression; we found that expression of c564-*Gal4* was enhanced by *fs(1)h* knockdown, but *foxo* heterozygosity had no effect on *Gal4* expression (Fig. S3A).

We next tested the effect of *foxo* heterozygosity on AMP expression in *fs(1)h* knockdowns. We found that AMP expression was almost entirely normalised in uninfected *fs(1)h*-knockdown animals also lacking one copy of *foxo* (Fig. 3B), in accordance with the known ability of FOXO to promote AMP expression (Becker et al., 2010). However, AMP expression was still significantly elevated in these animals following bacterial infection (Fig. 3C, Fig. S3B). Together, these data indicate that *fs(1)h* affects baseline and infection-induced AMP expression via distinct mechanisms: baseline AMP overexpression in *fs(1)h* knockdowns likely results from FOXO hyperactivation, while infection-induced AMP overexpression results from some other mechanism, possibly the observed overexpression of REL.

We then examined the combined effect of *foxo* and *fs(1)h* on metabolic phenotypes. *foxo* heterozygosity increased total triglyceride levels on an otherwise wild-type background; this phenotype was unaltered by *fs(1)h* knockdown, but *fs(1)h* knockdown animals lacking one copy of *foxo* regained their ability to utilise stored triglyceride (Fig. 3D). Levels of trehalose and glucose were also improved in *fs(1)h* knockdowns also lacking one copy of *foxo*, while glycogen levels were independent of *foxo* genotype (Fig. 3E). Finally, we tested the ability of our flies to survive starvation. As expected from their defect in triglyceride catabolism, *fs(1)h* knockdowns were markedly short-lived when starved; this effect was also ameliorated by *foxo* heterozygosity, in keeping with the improved ability of these animals to utilise stored triglyceride (Fig. S3C).

The strong phenotypic rescue we observed in adult flies heterozygous for *foxo* suggested that *fs(1)h* knockdown could also cause insulin-pathway loss-of-function phenotypes during development that might be visible as a change in adult size. We tested this by measuring wing size and total dry mass in *fs(1)h* knockdowns. We found that animals with *fs(1)h* knocked down in the fat body exhibited a non-significant trend toward reduced dry mass, accompanied by a small increase in wing size (Fig. S3D,E). These data suggest that the effect of fat-body *fs(1)h* knockdown on systemic insulin activity is not sufficient to strongly alter larval growth.

### FOXO regulates AKT activation state downstream of *fs(1)h*

**Fig. 4.**
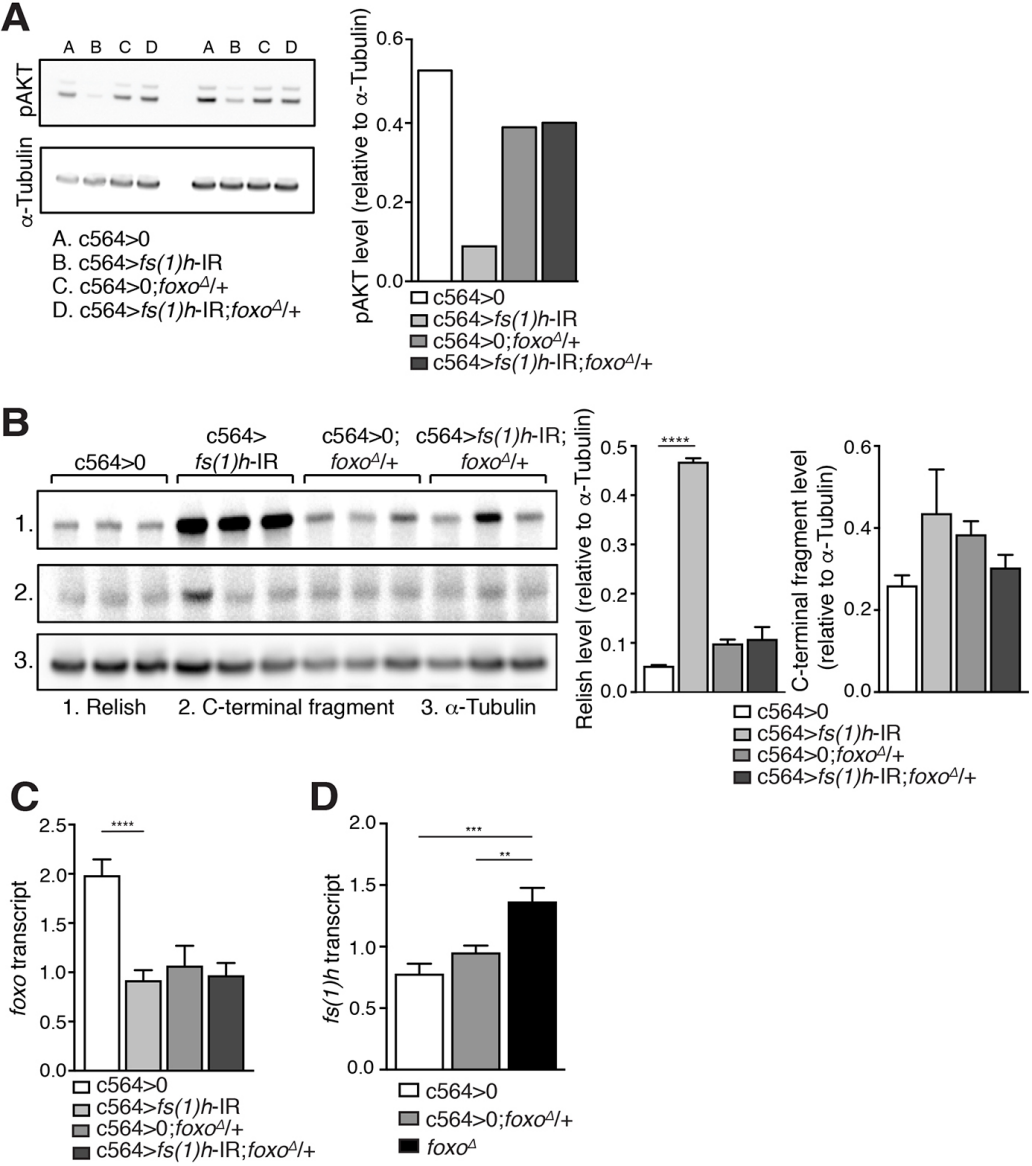
**FOXO regulates AKT activity downstream of *fs(1)h*.** (A) Western blot analysis of AKT Ser505 phosphorylation in control and *fs(1)h* fat-body-knockdown flies, either wild-type or heterozygous for *foxo*. Values represented as intensity relative to α-tubulin, shown as mean. (B) Western blot analysis of total and cleaved REL abundance in control and *fs(1)h* fat-body-knockdown flies, either wild-type or heterozygous for *foxo*. Values represented as intensity relative to α-tubulin, shown as mean+s.e.m.; genotypes were compared using unpaired two-tailed *t*-test. (C) *foxo* mRNA expression in control and *fs(1)h* fat-body-knockdown flies, either wild-type or heterozygous for *foxo*. Normalised to expression of α-tubulin as a loading control. Values shown as mean+s.e.m.; genotypes were compared using unpaired two-tailed *t*-test. (D) *fs(1)h* expression in flies wild-type, heterozygous or homozygous for *foxo* mutation. Normalised to expression of α-tubulin as a loading control. Values shown as mean+s.e.m.; genotypes were compared using unpaired two-tailed *t*-test. Throughout, **P<0.01, ***P<0.001, ****P<0.0001.

To establish the precise nature of the genetic interaction between *foxo* and *fs(1)h*, we tested the effect of *foxo* heterozygosity on AKT activation in *fs(1)h* knockdowns. We found that *foxo* heterozygosity was sufficient to rescue systemic AKT phosphorylation to normal levels (Fig. 4A). Not unexpectedly, insulin sensitivity was also essentially normal in these animals (Fig. S4A). REL protein levels were also normalised by *foxo* heterozygosity (Fig. 4B), in keeping with the normalisation of uninfected AMP expression in these animals.

We next examined the effects of *foxo* and *fs(1)h* on one another's expression. We found that *fs(1)h* knockdown reduced *foxo* transcript levels significantly, an effect that was unaltered by compound *foxo* heterozygosity (Fig. 4C). In turn, while *foxo* heterozygosity did not alter *fs(1)h* expression, *foxo*-null mutants expressed *fs(1)h* at significantly higher levels than wild-type controls (Fig. 4D). Together, these results suggest that the strong epistatic relationship between these genes reflects a fundamental functional connection.

Overall, our data indicate that the biological consequences of *fs(1)h* loss-of-function in the fat body are primarily mediated via effects on systemic regulation of physiological signalling, and that these effects are largely dependent on *foxo*.

## DISCUSSION

Here, we have shown for the first time that *fs(1)h*, encoding the sole *Drosophila* member of the BET bromodomain protein family, has critical functions in both immunity and metabolism: it is required for normal utilisation of triglyceride stores, to restrain expression of AMPs and to enable normal survival. Remarkably, it appears that almost all of these functions are due to effects on signalling via AKT and FOXO. *fs(1)h* knockdowns exhibited significant reductions in baseline AKT phosphorylation. Removal of a single copy of *foxo* completely rescued the short lifespan of *fs(1)h* knockdown animals, restored their ability to utilise triglyceride stores, and normalised their survival during starvation and their expression of AMPs when uninfected. Importantly, AMP expression was still significantly enhanced in infected *fs(1)h* knockdown *foxo* heterozygotes relative to controls, indicating that not all *fs(1)h* phenotypes can be attributed to *foxo* hyperactivation.

The overall physiological phenotype of *fs(1)h* knockdown is surprising, given what is known about insulin-AKT signalling functions in vertebrates and wild-type flies. Normally, we would expect impaired AKT activity to primarily impair anabolic processes; the effects we see on catabolism are unexpected. We suggest that the actual observed phenotype is a complex combination of developmental and physiological effects – flies with lifelong loss of insulin signalling exhibit increased adiposity (Tatar et al., 2001), while those with adult-specific loss of insulin signalling exhibit either normal triglyceride levels or are lean, depending on diet (Al Saud et al., 2015). Nonetheless, the effect of *fs(1)h* knockdown on AKT phosphorylation, and the ability of *foxo* mutation to completely rescue most phenotypes, suggest that *fs(1)h* may play an important and direct physiological role as a regulator of signalling via AKT.

The immune effects of *fs(1)h* knockdown and the interaction with *foxo* appear more straightforward. *foxo* is able to activate AMPs directly, but it is currently not believed to be an important regulator of AMPs in the fat body during infection (Becker et al., 2010). We and others have observed that bacterial infection and immune activation in *Drosophila* can drive disruption of systemic insulin signalling (DiAngelo et al., 2009; Dionne et al., 2006; Roth et al., 2018; Woodcock et al., 2015). Conversely, flies with genetic disruption of insulin signalling or fed high-sugar diets exhibit significant alterations in immune competence (Libert et al., 2008; Musselman et al., 2017, 2018). We have described this effect as, in part, a consequence of functional transcriptional switching in the fat body between metabolic and immune activities (Clark et al., 2013). Infection-induced AMP expression is the product of concerted signalling via NF-κB and MEF2 combined with tissue identity signals from GATA factors (Clark et al., 2013; Petersen et al., 1999; Senger et al., 2006). Together, our data and these other observations suggest that *fs(1)h* is normally suppressive on AMP loci no matter what the relevant activating cue. Reduction of *fs(1)h* permits AMP expression, which is promoted by *foxo* activity in the absence of infection; AMP expression is still stronger after infection, when the relevant activating signal is provided by NF-κB activity. Further work will be required to determine whether FS(1)H is a true nodal point between immune and metabolic signalling, or is a more general regulator of gene expression that happens to affect both processes.

In recent years, several drugs targeting interactions of epigenetic regulators with modified histones have been advanced into clinical trials, including drugs specifically targeting BET proteins. These compounds are of particular interest in the treatment of cancer and inflammatory disease, based in part on identification of transcriptional targets that are disproportionately dependent on BET inhibition (Shi and Vakoc, 2014). However, various pathological effects have been noted from genetic inhibition of BRD4 in mice (Bolden et al., 2014). Our data suggest that BET inhibition *in vivo* may have significant metabolic consequences via effects on AKT-FOXO signalling, as well as suggesting that AKT may be a key pharmacological target of BET inhibition in anti-tumour therapy. Potential metabolic roles of *Brd3* and *BrdT* have not been reported. However, mice lacking *Brd2* exhibit some metabolic similarities with *fs(1)h* knockdown flies, while BRD4 inhibits autophagy in cultured human cells and in the mouse intestine (Sakamaki et al., 2017; Wang et al., 2009), implying that aspects of the phenotype that we observed may be conserved in mammals.

## MATERIALS AND METHODS

### *Drosophila* stocks and culture

Fly stocks were maintained on food containing 10% w/v Brewer's yeast, 8% fructose, 2% polenta, 0.8% agar at room temperature or 25°C. Male flies were used for all experiments, always at 5- to 7-days old, collected for experiments as late as possible but when more than 85% of a given population was still alive. The following fly lines were used in this study (if not otherwise noted, obtained from Bloomington Drosophila Stock Center):

*w^1118^*;

*w^1118^*; c564-Gal4;

*w^1118^*; r4-Gal4 / TM6c, *Sb^1^*;

*w*; UAS-*fs(1)h*-IR (VDRC KK108662) (*fs(1)h* knockdown line I);

*w*; UAS-*fs(1)h*-IR (VDRC GD51227) (*fs(1)h* knockdown line II);

*w*; UAS-*bcd*-IR (VDRC KK104160) (*bcd* knockdown line); and

*w*; *foxo^Δ94^*/TM6c, *Sb^1^* (courtesy of Cathy Slack, Aston University, UK).

### Bacterial infection

*Listeria monocytogenes* EGD-E and *Francisella novicida* U112 were used for infections. Broth cultures were grown overnight at 37°C, either still (*L. monocytogenes*) or shaking (*F. novicida*)*.* The following day, the cultures were centrifuged at 4000 ***g*** for 10 min. The bacterial pellets were resuspended in sterile PBS and the optical density was measured at 600 nm (OD600). *Listeria monocytogenes* and *F. novicida* were injected at OD600=0.1. Infections were then performed using a pulled glass needle as previously described (Dionne et al., 2003).

### Survival assays

Male flies were collected following eclosion for 3-4 days to obtain between 20-30 age-matched flies per genotype. In all survival assays, each vial was checked daily and the number of dead flies was recorded. Food vials were kept horizontally during the assay to prevent the flies getting stuck in the food. For starvation-resistance experiments, flies were kept in vials containing 1% agar+2% PBS to prevent desiccation. All survival analyses are shown as single representative experiments; all survival analyses were performed at least three times independently.

### Smurf assay

Standard fly food was dyed using Blue dye no.1 (SPS Alfachem) at a concentration of 2.5% (wt/vol). Flies were maintained at 29°C on the dyed food for a 2-h time period in the morning. Flies were counted as a Smurf when the dye colouration could be observed outside of the digestive tract.

### qRT-PCR

RNA was extracted from groups of two to three flies using TRI reagent (Sigma) following the manufacturer's instructions. cDNA synthesis was carried using RevertAid Reverse Transcriptase and primed with random hexamers (Thermo Scientific). PCR was carried out with Sensimix SYBR Green no-ROX (Bioline) on a Corbett Rotor-Gene 6000. For all experiments, the cycling conditions used were as follows: hold 95°C for 10 min, then 40 cycles of 95°C for 15 s, 57°C for 30 s, 72°C for 30 s. Gene abundances were calculated based on experimental measurements on a standard curve; standards were run in every experiment, both to allow quantification and to verify that all measured values fell within the linear amplification range of the primer set. All measured mRNA abundances were first normalised to the housekeeping gene, *α-Tubulin*, and then analysed further. The graphed values are these tubulin-normalised abundance measurements. The qRT-PCR primers used in this study are shown in Table S1.

### Thin-layer chromatography (TLC)

Groups of ten flies were homogenised in 100 μl chloroform:methanol (3:1; both Sigma-Aldrich). A set of standards were prepared using lard (Sainsbury's Supermarkets Ltd) to generate a standard curve. Standards (2 μl per standard) and samples (20 μl per sample) were then loaded onto a TLC plate and migrated in a 4:1 mix of hexane:diethyl ether (both Sigma-Aldrich). TLC plates were stained using the general oxidising stain, ammonium heptamolybdate, left to dry and baked at 80°C for approximately 20 min. The plate was imaged using a Bio-Rad Molecular Imager and signal was quantitated using Bio-Rad Image Lab software.

### Fat-body imaging

Flies were fixed for 1 h 30 min in 4% paraformaldehyde (PFA) and 0.1% Triton X-100. Samples were then washed with 1× PBS and 0.1% Triton X-100. DAPI (Life Technologies; 1:1000) was added and incubated for 10 min at room temperature. The samples were washed with 1× PBS, and incubated with HCS LipidTOX Deep Red (Life Technologies; 1:200) in 1× PBS for 2 h at room temperature in the dark. Finally, the samples were mounted on glass coverslips in Fluoromount-G (eBioSciences). Images were acquired using a Leica SP5 microscope.

### Adult brain imaging

Brains (c564>*fs(1)h*-IR, *n*=8; c564>0, *n*=5) were dissected from adult male flies starved for 1 h into 1× PBS. Dissected brains were fixed in 4% formaldehyde (methanol-free) 0.1% Triton X-100 PBS (PBST) for 30 min at room temperature. Samples were then rinsed twice with 0.1% PBST and washed four times for 1 h. 5% normal goat serum in 0.1% PBST was used as a blocking agent for 1 h at room temperature. Brains were then incubated overnight at 4°C with primary antibodies in 5% normal goat serum in 0.1% PBST. The primary antibodies used were: anti-rabbit-dILP2 (gift from Nazif Alic, University College London, London, UK; 1:1000) and anti-Rat-Elav (DSHB #7E8A10; 1:1000). Brains were rinsed twice with PBST, and washed four times with 0.1% PBST for 1 h. Secondary antibodies were diluted in 5% normal goat serum in 0.1% PBST and incubated with the brains for 1 h at room temperature. The secondary antibodies used were: anti-rabbit-Alexa-Fluor-488 (Thermo Scientific #A27034; 1:500) and anti-rat-Alexa-Fluor-555 (Thermo Scientific #A21434; 1:500). Samples were then rinsed twice with 0.1% PBST, and washed four times for 1 h. Brains were mounted on glass coverslips in Vectashield (Vector Laboratories). All incubations and washes were performed in a rotator. Images were acquired using a Zeiss LSM 510 confocal microscope and edited using Fiji/ImageJ.

### Glucose/trehalose/glycogen assay

Flies were starved for 1 h, then homogenised on ice 75 μl in 10 mM Tris, pH 7.6; 1 mM EDTA+0.1% Triton X-100 in groups of three flies per genotype. The samples were heated for 20 min at 90°C to inactivate endogenous trehalase. Glucose was then measured using a glucose-oxidase-based enzymatic assay kit (Sentinel Diagnostics); the assay reagent was supplemented with amyloglucosidase to measure glycogen or trehalase to measure trehalose.

### Western blot

Western blotting was carried out as previously described (Clark et al., 2013). Primary antibodies used were anti-phospho-Akt (CST #4054; 1:1000), anti-Relish-C (DSHB #21F3; 1:1000) and mouse anti-α-tubulin (DSHB 12G10; 1:10,000). Secondary antibodies were horseradish peroxidase (HRP)-conjugated anti-rabbit-IgG, 1:5000 and HRP-conjugated anti-mouse IgG, 1:5000, both from Cell Signaling Technologies. Monoclonal anti-REL and anti-tubulin were obtained from the Developmental Studies Hybridoma Bank, created by the NICHD of the NIH and maintained at The University of Iowa, Department of Biology, Iowa City, IA, USA.

### Insulin-sensitivity experiment

Flies were fasted for 1 h on 1% agar, then injected with 50 nl of sterile PBS (vehicle) or human insulin in PBS at a low dose (1.28 μg/ml) or a high dose (6.4 μg/ml). At 10 min post-injection, flies were homogenised in groups of three in 75 μl of 2× Laemmli loading buffer; these lysates were then assayed for AKT activity by western blot.

### Statistical analysis

For qRT-PCR, TLC and the glucose/trehalose/glycogen assay, standards were used to calculate values for each sample. The qRT-PCR analysis was also relative to α-tubulin as housekeeping control, and western blot analysis was relative to the α-tubulin loading control. Western blot and TLC images were exposed and the band densities were analysed using ImageJ software. Unpaired *t*-tests or two-way ANOVA were used to calculate statistical significance for all experiments. For survival experiments, the log-rank and Wilcoxon tests were carried out in GraphPad Prism.

### Wing measurements

Fly wings were mounted on glass slides using glycerol and photographed. Areas were calculated using ImageJ. Ten wings were measured per genotype.

### Fly dry masses

A total of 80 flies were collected per genotype, killed by freezing, desiccated using silica gel in a closed container at room temperature for 1 week, then weighed.

## Supplementary Material

Supplementary information
