# Supplementary material for: fs(1)h controls metabolic and immune function and enhances survival via AKT and FOXO in Drosophila
Source: Dis Model Mech. 2019 Apr 4;12(4):dmm037259. doi: 10.1242/dmm.037259 (PMC6505478; doi:10.1242/dmm.037259)
Supplement: Supplementary information [file dmm-12-037259-s1.pdf]

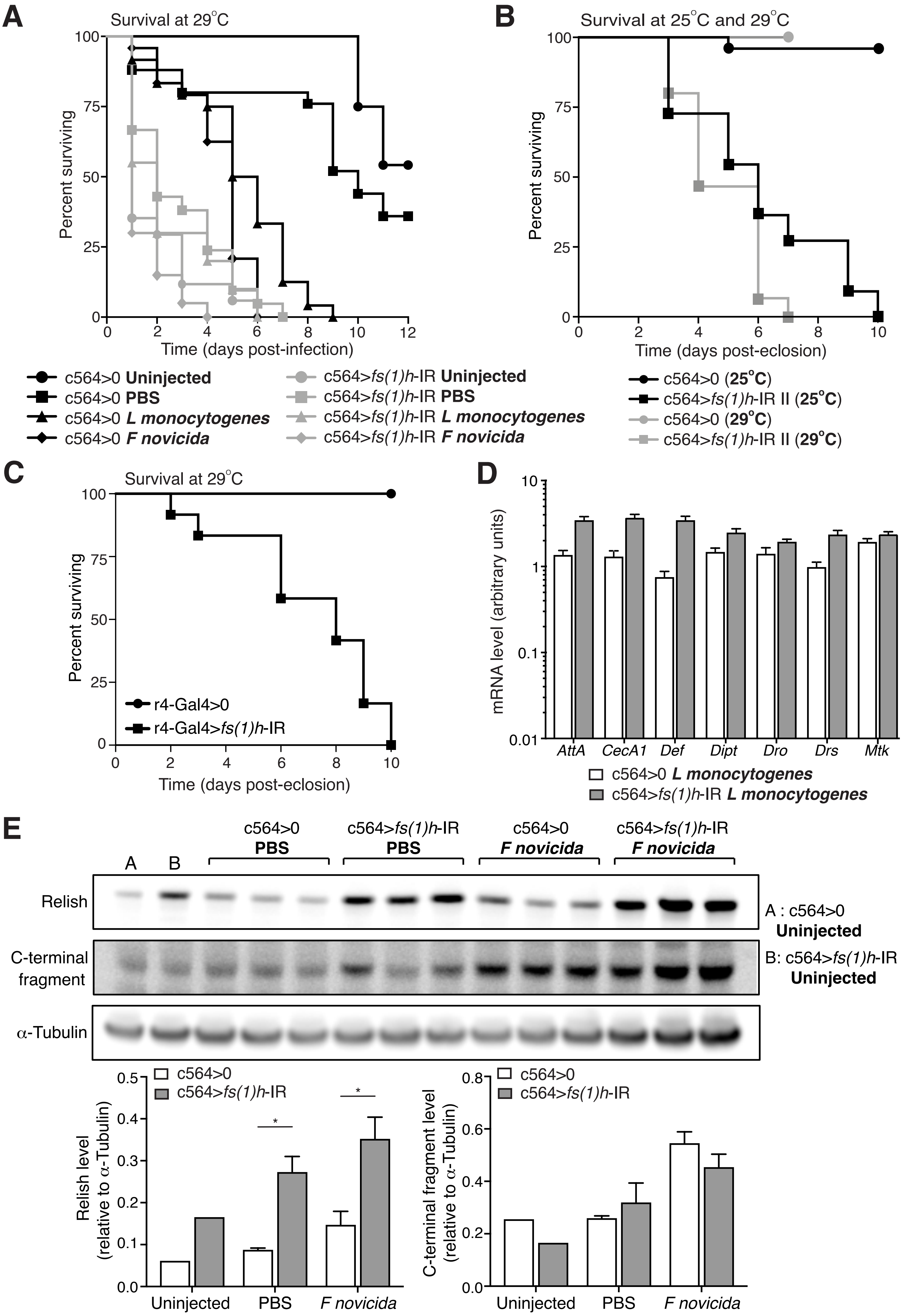

Disease Models & Mechanisms • Supplementary information

Figure S1. (A) Survival of fat body knockdown of *fs(1)h* (KK108662) compared to controls, either uninfected, PBS injected, or infected with *L monocytogenes* or *F novicida.* (B) Survival of flies with fat body knockdown of *fs(1)h* using a different RNAi line (GD51227) compared to driver-only controls. (C) Survival of flies with *fs(1)h* knocked down in fat body under the control of r4-Gal4. (D) Expression of antimicrobial peptide (AMP) genes in *Listeria monocytogenes*-infected 5-7 day old male control (c564>0) and *fs(1)h* fat body knockdown (c564>*fs(1)h-IR*) flies. Values shown as mean + SEM. (E) Relish protein levels in *fs(1)h* knockdowns and control animals, uninfected, PBS-injected, or infected with *Francisella novicida*.

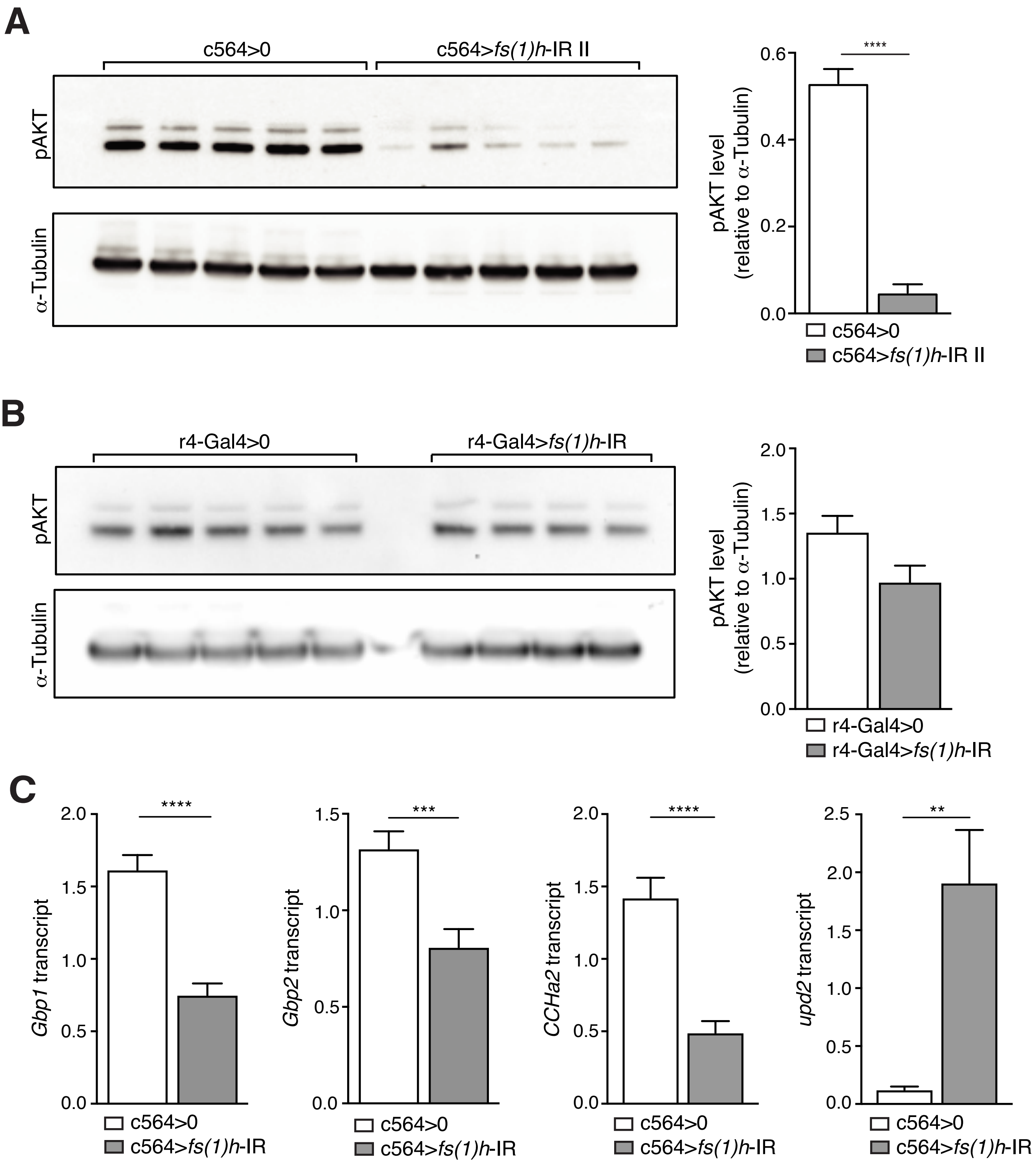

Figure S2. (A) Western blot analysis of AKT phosphorylation in control and *fs(1)h* (GD51227) fat body knockdown flies. Values represented as intensity relative to α-Tubulin, shown as mean+SEM, genotypes were compared using unpaired two-tailed t-test (****p<0.0001). (B) Western blot analysis of AKT phosphorylation in flies with *fs(1)h* knockdown driven by r4-Gal4. Values represented as intensity relative to α-Tubulin. (C) Expression of insulin-regulatory secreted factors in isolated fat body from control and *fs(1)h* fat body knockdown flies, shown as mean + SEM. Normalised to expression of α-tubulin as a loading control.

Disease Models & Mechanisms • Supplementary information

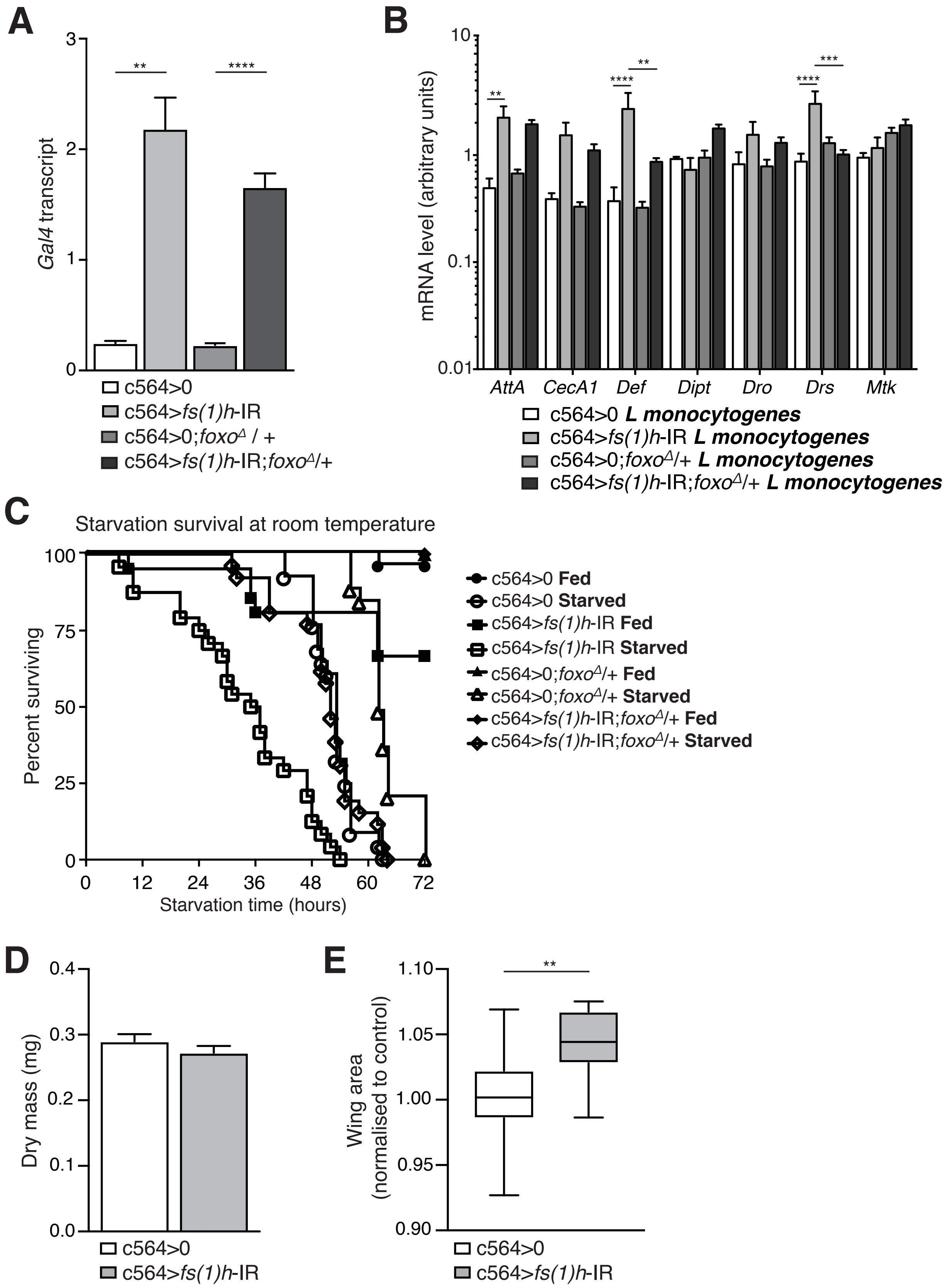
A
Gal4 transcript
3
2
1
0
**
****
c564>0
c564>fs(1)h-IR
c564>0;foxoΔ / +
c564>fs(1)h-IR;foxoΔ/+
B
mRNA level (arbitrary units)
10
1
0.1
0.01
**
****
**
****
***
AttA
CecA1
Def
Dipt
Dro
Drs
Mtk
c564>0 L monocytogenes
c564>fs(1)h-IR L monocytogenes
c564>0;foxoΔ/+ L monocytogenes
c564>fs(1)h-IR;foxoΔ/+ L monocytogenes
C
Starvation survival at room temperature
Percent surviving
100
75
50
25
0
0
12
24
36
48
60
72
Starvation time (hours)
c564>0 Fed
c564>0 Starved
c564>fs(1)h-IR Fed
c564>fs(1)h-IR Starved
c564>0;foxoΔ/+ Fed
c564>0;foxoΔ/+ Starved
c564>fs(1)h-IR;foxoΔ/+ Fed
c564>fs(1)h-IR;foxoΔ/+ Starved
D
Dry mass (mg)
0.4
0.3
0.2
0.1
0.0
c564>0
c564>fs(1)h-IR
E
Wing area (normalised to control)
1.10
1.05
1.00
0.95
0.90
**
c564>0
c564>fs(1)h-IR

Figure S3. (A) *Gal4* expression in *fs(1)h* knockdowns and controls, wild-type or heterozygous for *foxo*. Data shown as mean + SEM; genotypes were compared using unpaired two-tailed t-test (**p<0.01, ****p<0.0001). (B) Expression of antimicrobial peptide (AMP) genes in *Listeria monocytogenes*-infected 5-7 day old male control (c564>0) and *fs(1)h* fat body knockdown (c564>*fs(1)h-IR*) flies, either wild-type or heterozygous for *foxo*. Values shown as mean + SEM. (C) Starvation survival of control and *fs(1)h* fat body knockdown flies, either wild-type or heterozygous for *foxo*. Carried out at room temperature on 5-7 day old flies. (D) Dry mass per fly of control and *fs(1)h* fat body knockdown animals. Values shown are mean + SEM. (E) Wing areas of control and *fs(1)h* fat body knockdown animals, normalised to the mean of the controls. The center line represents the median of the measurement; the extent of the box, the interquartile range; the whiskers, the furthest possible outliers. **p<0.01 by Wilcoxon rank-sum test.

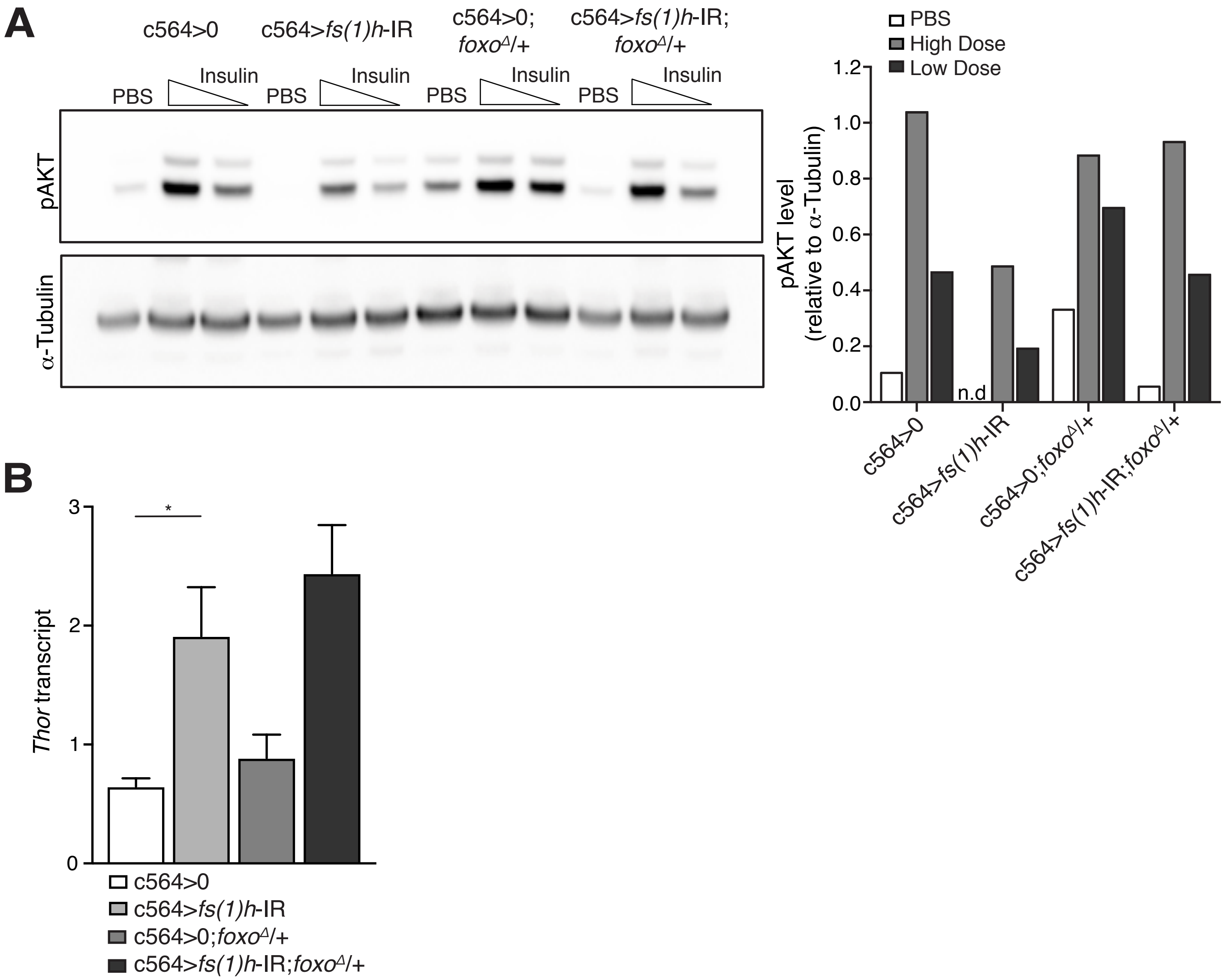

Figure S4. (A) Western blot analysis of AKT phosphorylation following injection with PBS or a high (320 pg/fly) or low (64 pg/fly) dose of human insulin. Values relative to α-Tubulin, shown as mean. (B) Expression of *Thor* in control and *fs(1)h* knockdown flies, either heterozygous or wild-type for *foxo*.

Table S1: qRT-PCR primers used in this study:

**Gene**	**Left**	**Right**
AttA	CACAATGTGGTGGGTCAGG	GGCACCATGACCAGCATT
Bmm	GTCTCCTCTGCGATTTGCCAT	CTGAAGGGACCCAGGGAGTA
CCHa2	TGCTACTGGTCGTTATCTGCAC	TAGGCCTGGCATCCCTTT
CecA1	TCTTCGTTTTCGTCGCTCTC	CTTGTTGAGCGATTCCCAGT
CG10702	GGACTCCCTGCAGACCAA	AGACCGATGGGTTCGTTTCT
conv	TTAGGGGTATGCGTTTCCTC	GACCAATATCCGTGAGCACAT
Def	TTCTCGTGGCTATCGCTTTT	GGAGAGTAGGTCGCATGTGG
Dipt	ACCGCAGTACCCACTCAATC	CCCAAGTGCTGTCCATATCC
Dro	CCATCGAGGATCACCTGACT	CTTTAGGCGGGCAGAATG
Drs	GTACTTGTTCGCCCTCTTCG	CTTGCACACACGACGACAG
foxo	ACTTTCCCGTGGGCAATC	CGCCGTAGCTCATCTCCT
Gal4	GCAGCATTCTGGAACAAAGA	GGACAATTGGATCTCCCAAG
Gbp1	CAGCACAGAACAGATCACCAG	GCAGCACAGTGGTGTTTTCA
Gbp2	AAACGGCGGACATGGATA	TGGAAAACGACCGAAGACA
Hnf4	AGGAAATGTTGTTGGGAGGA	GGGTGGGACTCTGGTAGTCA
Hsl	CTTGGAAATACTTGAGGGGTTG	AGATTTGATGCAGTTCTTTGAGC
ImpL2	GCCGATACCTTCGTGTATCC	TTTCCGTCGTCAATCCAATAG
Mtk	TCTTGGAGCGATTTTTCTGG	TCTGCCAGCACTGATGTAGC
Plin1	GCGTTCTATGGTAGCCTTCAG	GCGTCCGGATAGAAAGCTG
Plin2	GCAGAATGGCAAGAGTTCTGA	ACTGTGTGTAGGACTGGATCCTC
Sdr	GCAGCACTTTTCCCTTAAACA	GGGCGAAGTTGTGTCGTATT
Thor	CAGGAAGGTTGTCATCTCGGA	GGAGTGGTGGAGTAGAGGGTT
upd2	CGGAACATCACGATGAGCGAAT	TCGGCAGGAACTTGTACTCG
α-Tubulin	GATTCCGGTGACGGTGAG	GATGCACACCTATCCGCTCT

Disease Models & Mechanisms • Supplementary information
